# Stress-inducible expression of *AtDREB1A* transcription factor greatly improves drought stress tolerance in transgenic* indica* rice

**DOI:** 10.1007/s11248-013-9776-6

**Published:** 2014-01-08

**Authors:** G. Ravikumar, P. Manimaran, S. R. Voleti, D. Subrahmanyam, R. M. Sundaram, K. C. Bansal, B. C. Viraktamath, S. M. Balachandran

**Affiliations:** 1 Directorate of Rice Research, Rajendranagar, Hyderabad, 500 030 India; 2 National Bureau of Plant Genetic Resources, Pusa Campus, New Delhi, 110 012 India

**Keywords:** Drought stress tolerance, *AtDREB1A* gene, Transgenic rice, Transcription factors

## Abstract

The cultivation of rice (*Oryza sativa* L.), a major food crop, requires ample water (30 % of the fresh water available worldwide), and its productivity is greatly affected by drought, the most significant environmental factor. Much research has focussed on identifying quantitative trait loci, stress-regulated genes and transcription factors that will contribute towards the development of climate-resilient/tolerant crop plants in general and rice in particular. The transcription factor *DREB1A*, identified from the model plant *Arabidopsis thaliana*, has been reported to enhance stress tolerance against drought stress. We developed transgenic rice plants with *AtDREB1A* in the background of* indica* rice cultivar Samba Mahsuri through *Agrobacterium*-mediated transformation. The *AtDREB1A* gene was stably inherited and expressed in T_1_ and T_2_ plants and in subsequent generations, as indicated by the results of PCR, Southern blot and RT-PCR analyses. Expression of *AtDREB1A* was induced by drought stress in transgenic rice lines, which were highly tolerant to severe water deficit stress in both the vegetative and reproductive stages without affecting their morphological or agronomic traits. The physiological studies revealed that the expression of *AtDREB1A* was associated with an increased accumulation of the osmotic substance proline, maintenance of chlorophyll, increased relative water content and decreased ion leakage under drought stress. Most of the homozygous lines were highly tolerant to drought stress and showed significantly a higher grain yield and spikelet fertility relative to the nontransgenic control plants under both stressed and unstressed conditions. The improvement in drought stress tolerance in combination with agronomic traits is very essential in high premium* indica* rice cultivars, such as Samba Mahsuri, so that farmers can benefit in times of seasonal droughts and water scarcity.

**Electronic supplementary material:**

The online version of this article (doi:10.1007/s11248-013-9776-6) contains supplementary material, which is available to authorized users.

## Introduction

Drought is the most significant environmental stress on agricultural production worldwide (Cattivelli et al. 2008), and a tremendous effort is being applied to improve crop yields in the face of increasing water scarcity. Rice (*Oryza sativa* L.) is the most widely consumed food crop and is grown on 160 million hectares worldwide (FAO 2007). Globally, more than 3 billion people from Asia and other countries depend on rice as their staple food, and by 2025 about 60 % more rice must be produced to meet the needs of the growing population. Drought affects plant growth, yield, membrane integrity, pigment content, osmotic adjustments, water relations and photosynthetic activity (Benjamin and Nielsen 2006). Drought-prone regions and potential agricultural land with no irrigation system in place have been less exploited than those with developed irrigation systems or more reliable rainfall due to difficulties and high costs of developing improved technologies. As a result, rice yields are showing a steady decrease worldwide in unirrigated and drought-prone areas. Therefore, developing drought-tolerant rice varieties and reducing water consumption during rice production is crucial to increased rice yield.

Due to the complex polygenic nature of drought tolerance, attempts to improve this trait through conventional breeding have met with little success. Alternatively, the identification and transfer of genes that confer resistance/tolerance to drought stress through transgenic technology is often projected as one solution for protecting crops against a water stress environment and increasing crop yields worldwide, particularly in less developed areas that are threatened by food scarcity and low crop productivity (Nelson et al. 2007). The transgenic approach involves structurally modifying traits by transferring desired genes from one species to other (Ashraf 2010) without any barrier, and it has been employed to overexpress genes from the model dicotyledonous plant *Arabidopsis* to many crop plants.

Transcription factors (TFs: activators and repressors) are key regulators of the changes in gene expression and environmental stress responses. They have been proved to be useful for improving plant stress tolerance through inducing the expression of a number of stress-related target genes (Thomashow 2001). Both transcription activators and repressors have been shown to confer drought stress tolerance (Abe et al. 2003; Sakuma et al. 2006). Most of such TFs have been identified and analysed in *Arabidopsis,* in which genome-wide microarray analyses have helped to identify several potential target genes (Bray 2004; Denby and Gehring 2005; Shinozaki et al. 2003). The best characterized TF groups are ABA responsive element binding protein1 (AREB1), ABA responsive binding factor 2 (ABF2), dehydration-responsive binding protein (DREB) genes, MYB genes, bZIP encoding genes and the protein kinases such as receptor like kinase 1, SNF1-related protein kinase 2C or guard cell expressing calcium dependent protein kinases (Choi et al. 2000; Osakabe et al. 2005; Umezawa et al. 2004; Uno et al. 2000). Many TF genes have been used to produce transgenic rice lines with either constitutive or inducible promoters, such as *HvCBF4* (Oh et al. 2007), *AP37* (Oh et al. 2009), *TaSTRG* (Zhou et al. 2009), ERF protein *TSRF1* (Quan et al. 2010), ERF protein *JERF3* (Zhang et al. 2010), *OsDREB2A* with the 4ABRC promoter (Cui et al. 2011), *OsDREB2A* with the *rd29A* promoter (Mallikarjuna et al. 2011), *SbDREB2* (Bihani et al. 2011), *OsSD1R1* (Gao et al. 2011), *OsDREB1A*, *OsDREB1B* and *AtDREB1A* (Datta et al. 2012).

The *Arabidopsis* gene *CBF3/DREB1A* has been used to improve abiotic stress tolerance in *japonica* rice (*Oryza sativa* cv. Nakdong) by constitutive expression (Oh et al. 2005) and in* indica* rice (*O. sativa* cv. BR29) by inducible expression (Datta et al. 2012). In the study reported here, we improved the drought tolerance of highly recalcitrant* indica* rice cultivar Samba Mahsuri (BPT 5204), which has medium slender grains with excellent cooking quality, by introducing the *Arabidopsis CBF3/DREB1A* gene driven by the stress-inducible *rd29A* promoter.

## Materials and methods

### Vector and gene construct

**Fig. 1:**
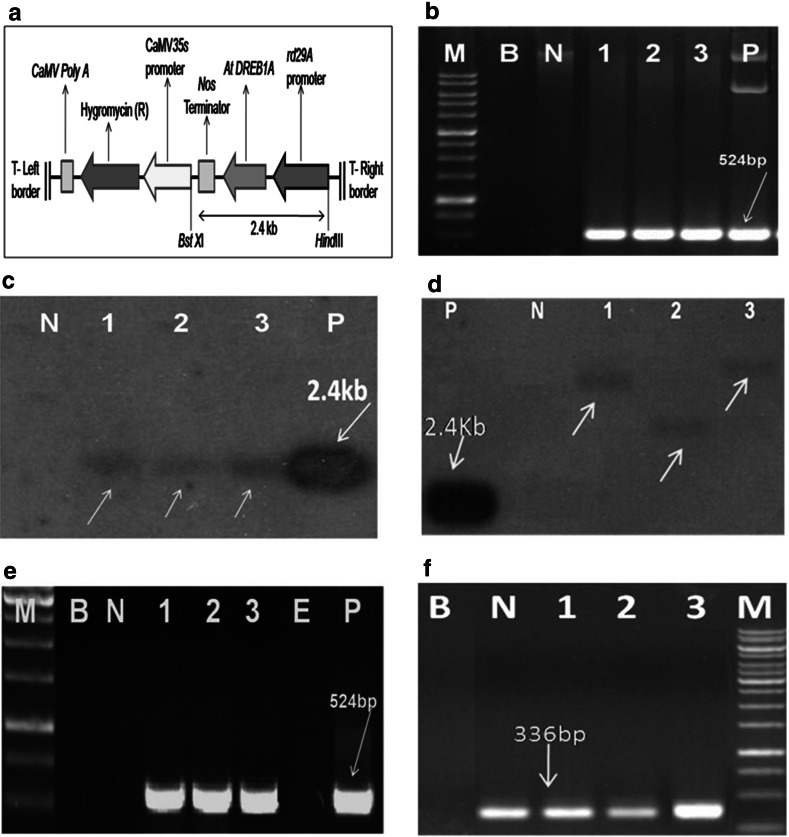
**a** Linear map of the expression cassette of the *AtDREB1A* gene driven by the *rd29A* promoter used for the transformation of rice variety BPT-5204 (Samba Mahsuri). **b** PCR analysis of primary T_0_ transgenic plants of BPT 5204 with gene-specific primers.* Lanes*: * M* 1-kb DNA ladder,* B* blank,* N* nontransformed control plants of BPT-5204,* 1–3* putative transgenic plants (*1* BD-33, *2* BD-38,* 3* BD-45),* P* positive control (plasmid). **c** Southern analysis of T_0_ BPT-dehydration responsive binding protein (DREB1A) plants with plasmid probe.* Lanes*: *N* Nontransformed control plants of BPT-5204, *1–3* putative transgenic plants (*1* BD-33, *2* BD-38, *3* BD-45),* P* positive control (2.4-kb eluted fragment of gene expression cassette). **d** Copy number detection of Southern-positive plants.* Lanes*:* P* positive control (plasmid),* N* nontransformed control plant,* 1–3* Southern-positive plants (*1* BD-33, *2* BD-38, *3* BD-45). **e** Reverse transcription (RT)-PCR analysis of primary T_0_ transgenic plants with gene-specific DREB primers (524 bp).* Lanes*:* M* 1-kb DNA ladder,* B* blank,* N* nontransformed control plant of BPT-5204, *1–3* putative transgenic plants (*1* BD-33, *2* BD-38, *3* BD-45),* E* empty,* P* positive control (plasmid). **f** RT-PCR analysis of primary T_0_ transgenic plants with *Actin* primers (336 bp) as internal controls.* Lanes*:* M* 1-kb DNA ladder,* B* blank,* N* nontransformed control plant, *1–3* putative transgenic plants (*1* BD-33, *2* BD-38, *3* BD-45)

*Agrobacterium tumefaciens* strain *LBA4404* carrying the binary vector pC1200-rd29A-AtDREB1A with a hygromycin-resistant gene (*hpt*) as plant selection marker and chloramphenicol as bacterial selection marker was used for transformation. The target gene (TF) was derived from *Arabidopsis thaliana* driven by the stress-inducible *rd29A* promoter and a *nos* terminator (Fig. 1a). Initially, the *Agrobacterium* culture was streaked on petri plates containing solid YEB medium (yeast extract 0.1 %, beef extract 0.5 %, sucrose 0.5 %, peptone 0.5 %, magnesium sulphate heptahydrate 0.05 %, agar–agar 1.5 %) containing 10 mg/l rifampicin, 25 mg/l chloramophenicol and incubated at 28 °C for 48 h. Starter cultures (5 ml) were obtained from the streaked plates and incubated in YEB broth supplemented with same antibiotics (5 μl) at 28 °C on a shaker at 220 rpm for 24 h.

### Plant material used for genetic transformation and selection of callus

Matured seeds of the* indica* rice cultivar Samba Mahsuri (BPT-5204) were used for the induction of embryogenic callus. Sterilized seeds were inoculated on MS (Murashige and Skoog 1962) medium supplemented with 2,4-dichlorophenoxyacetic acid (2,4-D; 2 mg/l) and kinetin (0.5 mg/ml) and incubated at 25 °C in the dark for callus induction. Embryogenic calli aged 15–21 days were selected under the microscope and subcultured on fresh MS medium for 5 days. *Agrobacterium* starter cultures incubated overnight were transferred to fresh YEB liquid medium and incubated at 28 °C for 6 h. At 1.0 OD, bacterial cells were harvested by centrifugation at 3,200 rpm for 20 min and re-suspended in MS medium containing 100 μM acetosyringone (MS-AS medium). Pre-incubation of bacterial suspensions was carried out at room temperature for 45 min, and the infection of embryogenic calli was done by vacuum infiltration for 20 min, following which all of the bacterial solution was removed. The calli were then co-cultivated on a sterile filter paper mat in co-cultivation medium supplemented with 200 μM AS for 3 days at 25 °C, following which the calli were washed three times, 15 min each wash, in sterile distilled water and liquid MS medium containing 250 mg/l cefotaxim and 250 mg/l carbenicillin antibiotics. All washed calli were selected on 50 mg/l hygromycin selection medium for three cycles of 15 days each cycle. Resistant calli were transferred onto regeneration medium containing kinetin (2 mg/l) and 1-naphthaleneacetic acid (NAA; 1 mg/l). Rooting of shoots/plantlets was done in half-strength MS medium in culture tubes. All of the plantlets thus derived were hardened in Yoshida’s culture solution and transplanted in earthen pots under biosafety glasshouse conditions. The protocols for callus induction, transformation, selection of transformed calli and regeneration of plantlets were as described in the DRR Laboratory Manual (Balachandran 2011).

### Identification and molecular characterization of transgenic rice plants

#### PCR analysis of putative transgenic plants

The molecular characterization of putative transgenic plants involved PCR and Southern blot analyses. For PCR, genomic DNA was isolated from a small piece of the leaves collected from putative transgenic plants at the hardening stage in Yoshida’s solution by using the CTAB method as described in the (DRR Laboratory Manual). A 10-μl PCR reaction volume was prepared with 50 ng genomic DNA isolated from leaves, 2.5 mM of dNTP, 1 U Taq DNA polymerase, 2 pM of primers and buffer with 1.5 mM MgCl_2_. The PCR reaction was set up with an initial denaturation at 94 °C for 5 min, followed by 35 cycles with denaturation at 94 °C for 30 s, annealing at 60 °C for 30 s, elongation at 72 °C for 1 min and a final extension step at 72 °C for 7 min. The amplified product was loaded onto a 1.2 % agarose gel and checked for amplified fragments of 524 bp. The primer sequences used were 5′AAGAAGTTTCGTGAGACTCG3′ for the forward primer and 5′CTCCATAACGATACGTCGTC3′ for the reverse primer.

#### Southern blot analysis

Southern blot analysis was carried out by isolating genomic DNA from 2 g of leaf tissue from both transgenic and nontransgenic control plants. About 20 μg of genomic DNA was digested with* Bst*X1 and* Hin*dIII for 16 h at 37 °C. Restricted samples were loaded onto 0.8 % agarose gel and subjected to electrophoresis overnight at 20 V. Restricted fragments in the gel were depurinated (0.025 M HCl) for 15 min, followed by denaturation (0.4 N NaOH) for 30 min and then transferred to nylon membrane by vacuum blotting for 20 min. Membranes were pre-hybridized for 6 h and then hybridized with probe DNA labelled with ^32^P in hybridization solution [0.5 M phosphate buffer pH 7.2, 14 % sodium dodecyl sulfate (SDS), 0.5 mM EDTA, 10 % bovine serum albumin (BSA) and 5 % SSS DNA] for 16 h. About 100 ng (45 μl) of eluted plasmid DNA was denatured and mixed with the beads (GE Healthcare, Little Chalfont, UK) containing dNTPs. A 5-μl aliquote of alpha^32^P was added to 45 μl of bead solution and incubated for 45 min at 37 °C. Labelled DNA was denatured and added to the hybridization solution containing the membranes. The next day blots were washed with Solution I (2× SSC, 0.1 % SDS) for 15 min, with Solution II (1× SSC, 0.1 % SDS) for 15 min and with Solution III (0.1× SSC, 0.1 % SDS) for 10 min. Washed blots were wrapped with saran wrap and loaded inside an X-ray film cassette and exposed for 24 h to detect signals. Further, copy number detection was made by restriction digestion of genomic DNA of transgenic and control plants with enzyme* Bst*X1 alone, which cuts at a unique site, followed by the same procedure for blotting and hybridization with probe DNA as described above.

#### Reverse transcriptase-PCR analysis

Southern- and PCR-positive transgenic plants were subjected to drought stress by withholding water for 7 days to study the expression of the *AtDREB1A* gene under the control of the stress-inducible promoter *rd29A*. Total RNA of the plants was extracted from the leaf tissue by using Trizol Reagent (Invitrogen, Carlsbad, CA). Reverse transcriptase (RT)-PCR was carried out using RNase-free DNase I-treated total RNA according to the RT PCR TM kit of Promega (Madison, WI). A 1-μg aliquot of total RNA from each sample was used in a 10-μl RT reaction volume using oligo dT as a primer; the cycling program consisted of 25 °C for 5 min, 42 °C for 45 min and 72 °C for 10 min. A 2-μl sample of the RT reaction volume after the completion of cDNA synthesis was used for amplification of *AtDREB1A* in a 10-μl PCR reaction volume. The same primers and procedure described above were used for PCR amplification of *AtDREB1A* cDNA from the transgenic plants, with the exception that 28 cycles were used instead of 35. During drought stress, the RT-PCR analysis was done on T_3_ homozygous lines with gene-specific *AtDREB1A* primers and *OsActin* primers as internal controls. The *Actin* primer sequences used were 5′-CCTTACCGACAACCTTATGA-3′ for the forward primer and 5′-ATGGAGTTGTATGTGGCTTC-3′ for the reverse primer.

#### Generation advancement and progeny analysis of transgenic plants

Fifty seeds collected from each of the three T_0_ Southern-positive plants were used to raise T_1_ plants for segregation studies in the T_1_ generation. All T_1_ plants were analysed by PCR to determine the inheritance pattern of the *AtDREB1A* gene. Southern analysis was also carried out for all 40 plants of each T_1_ line. All PCR- and Southern-positive T_1_ plants were harvested, and 25 seeds of each T_2_ plant were sown from 12 lines of BD-33, eight lines of BD-38 and seven lines of BD-45. PCR analysis of T_2_ plants of all three events was carried out, and the results confirmed integration of the target gene. The harvested T_3_ seeds from T_2_ homozygous lines (from all three events) were advanced further and confirmed the gene inheritance and homozygosity by PCR. These homozygous lines were selected for gene expression and evaluation studies against drought stress in the T_3_ generation.

### Evaluation of transgenic rice for drought tolerance

Evaluation of homozygous (T_3_) transgenic plants for expression of the targeted trait (drought stress) was carried out in hydroponic, vegetative and reproductive stages. Ten 4-week-old plants from each of five homozygous T_3_ lines and nontransformed control plants were subjected to an initial water stress treatment in Yoshida’s medium containing 20 % polyethylene glycol (PEG; hydroponics).

Homozygous plants and nontransformed controls were planted at the fifth leaf stage in the same type of pots containing homogenous soil composed of loam with a low level of organic matter; the pots were kept in a biosafety glasshouse. At 20 days post-transplantation, when the plants had nine to ten leaves, the control wild type (WT) and transgenic plants were divided into two groups. One group was maintained under well-watered normal conditions (unstressed arm) and the other group was subjected to drought stress by withdrawing water initially for 14 days inside the biosafety glass house and then sufficiently re-watered for 7 days. The highly tolerant plants (from the vegetative stage) were checked for drought stress tolerance also in the reproductive stage. During the reproductive stage, both transgenic and control plants were subjected to drought stress by withholding water for 14 days followed by re-watering. During the drought stress period, the water content of the soil was kept at about 15 % by restricting the amount of water applied. Agronomic traits, such as spikelet fertility, grain yield and days for maturity, were observed.

For the severe drought stress studies, homozygous transgenic (T_5_) lines, along with nontransgenic, drought-tolerant checks (Rasi, Vandana and N-22) and WT BPT controls were subjected to water deficit stress for 28 days in the vegetative stage. Similarly, the same lines were also subjected to drought stress for 15 days in the booting and anthesis stages along with nontransgenic controls. Another set of plants were screened in the real-time condition by planting them in soil in the biosafety screen house, and drought stress response was recorded after 30 days.

### Physiological studies

All physiological parameters were analysed at four stages, namely, before stress, 7 and 14 days of stress, and after re-watering. Relative water content (RWC) was measured by taking the fresh weight, turgid weight and dry weight of transgenic and control leaves. RWC was calculated from the equation of Schonfeld et al. (1988): ${\text{RWC }}\left( \% \right) = \left( {{\text{FW}} - {\text{DW}}} \right)/\left( {{\text{TW}} - {\text{DW}}} \right) \times 100$

A slightly modified method from that of Sullivan and Ross (1979) and Deshmukh et al. (1991) was followed for determining the percentage of ion leakage for both transgenic and control plants. Fully expanded leaves were collected from top second and third position from the main culm of both transgenic and control plants. Uniformly, 1-cm^2^ leaf bits totaling 10 cm^2^ in area were immersed in 25 ml distilled water, and the initial electrolyte conductivity (EC_a_) was measured. The leaf sample was then kept in the water bath at 50 °C for 1 h, and the conductivity (EC_b_) was measured. The contents were then incubated at ambient temperature overnight to complete ion leakage from tissues, and the electrolyte conductivity (EC_c_) was measured once again. The electrolyte leakage was calculated using the following formula:

$$ \% {\text{ Ion leakage}} = [\left( {{\text{EC}}_{\text{b}} - {\text{EC}}_{\text{a}} } \right)/{\text{EC}}_{\text{c}} ] \times 100 $$

Chlorophyll content was measured by using a soil and plant analysis device (SPAD) meter (Minolta Model 502; Minolta Co., Osaka, Japan) on leaves of both the transgenic and control plants. Proline content of the plants was estimated by the 3 % sulphosalysilic acid method described by Bates et al. (1973). The amount of the proline content was expressed in micrograms per gram plant FW.

Spikelet fertility and grain yield per plant were used as the major criteria to evaluate the drought resistance performance of each T_3_ homozygous transgenic line under biosafety glasshouse conditions. Spikelet fertility and grain yield were calculated by counting the total number of filled grains per panicle against total grains (filled and unfilled).

## Results

### Transformation

A total of 12,000 embryogenic calli induced on callus induction medium were infected by *Agrobacterium* strain LBA4404 carrying the binary vector pC1200-rd29A-*AtDREB1A* (Fig. 1a) in 15 different batches. Transformed calli were selected on media containing 50 mg/l hygromycin for three cycles of 15 days each. As many as 750 putative transgenic plants were regenerated from about the 2,500 resistant calli selected. All of the regenerated plantlets were hardened in Yoshida’s culture medium and maintained in transgenic biosafety glass house [Electronic Supplementary Material (ESM) Fig. S1].

### Molecular characterization

All of the 750 putative transgenic rice plants were screened by PCR using gene-specific primers; the 524-bp fragment, specific to the *AtDREB1A* gene, was amplified in 75 of these (Fig. 1b). These transgenic rice plants were further subjected to Southern hybridization analysis to confirm the integration of the *AtDREB1A* gene into the genome. Southern analysis clearly indicated the presence of hybridized bands of 2.4 kb from the *AtDREB1A* gene in three T_0_ transgenic plants, namely, BD-33, BD-38 and BD-45, thereby confirming the stable integration of the target gene (Fig. 1c). No hybridization signal was found in the nontransformed control plants. Determination of the copy number also revealed the presence of different hybridization signals, thereby confirming all three lines (BD-33, BD-38 and BD-45) as transgenic plants; these signals were confirmed to be indicative of single-copy insertions and independent events (Fig. 1d). The RT-PCR analysis involved the total RNA isolated from the three putative transgenic plants (BD-33, BD-38 and BD-45) and the control plants on days 3, 5 and 7 of the water stress treatment during the dry–down experiment in pots. The results of this analysis showed the amplification of the 524- and 366-bp fragments from the cDNAs of these three putative transgenic plants with the *DREB* primers (Fig. 1e) and *actin* primers, respectively (Fig. 1f), but not in the controls. All three Southern-positive plants (BD-33, BD-38 and BD-45) also showed gene expression as evidenced by RT-PCR (Fig. 1e).

### Generation advancement and progeny analysis of transgenic plants

**Fig. 2:**
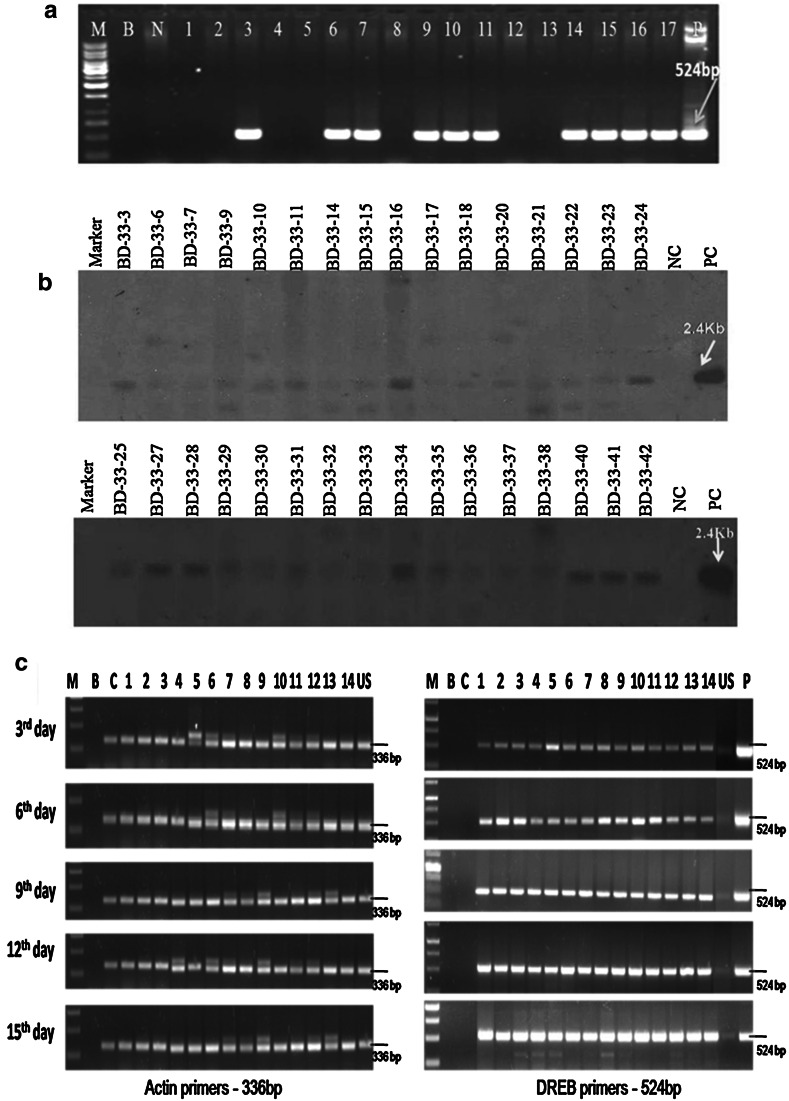
**a** PCR analysis of T_1_ BPT-DREB1A plants of the BD-33 line with gene-specific primers.* Lanes*:* M* 1-kb DNA ladder,* B* blank,* N* nontransformed control plant,* 1–17* T_1_ DREB plants (BD-33-1 to BD-33-17),* P* positive control (plasmid). **b** Southern analysis of T_1_ BPT-DREB1A plants of the BD-33 line with the plasmid probe.* Lanes*: * Marker* Lamda/*Hin*dIII digest,* BD-33-3–BD-33-42* BD-33-3 to BD-33-42 T_1_ plants,* NC* nontransformed control plant,* PC* positive control (2.4-kb eluted fragment of gene expression cassette). **c** Time course analysis of gene expression of T_3_ homozygous lines by RT-PCR with *Actin* (internal control) and gene-specific (*DREB*) primers during drought stress.* Lanes*:* M* 1-kb DNA ladder,* B* blank,* C* nontransformed control plant, *1–7* BD-33-24-4 lines (4-3, 4-4, 4-5, 4-6, 4-9, 4-10, 4-11), * 8–14* BD-33-24-5 lines (5-3, 5-4, 5-7, 5-8, 5-9, 5-10, 5-12),* US* unstressed transgenic plant,* P* positive control (plasmid)

Fifty seeds collected from each of the three T_0_ Southern-positive plants were used to raise T_1_ plants for segregation studies in the T_1_ generation. All of the T_1_ plants were analysed by PCR to determine the inheritance pattern of the *AtDREB1A* gene; 32 of the 42 BD-33 plants analysed were positive (Fig. 2a); 26 of the 40 BD-38 plants were positive and 30 of the 40 BD-45 plants were positive. Thus, PCR data on the individual lines showed a 3:1 segregation following the Mendelian ratio (ESM Table 1). Southern blot analysis was carried out for the T_1_ lines of the event BD-33, revealing that 32 of the 42 plants were positive (Fig. 2b). T_1_ transgenic lines of all three events that followed the Mendelian ratio of 3:1 were further advanced to the T_2_ generation. All PCR- and Southern-positive T_1_ plants were harvested, and 25 seeds each of the three T_2_ were sown from 12 lines of BD-33, eight lines of BD-38 and seven lines of BD-45. PCR analysis of the T_2_ plants of all three events confirmed the integration of the target gene, showing the presence of the 524-bp fragment among most of the transgenic lines but not in the controls (ESM Fig. S2a). The Southern blot analysis of BD-33 T_2_ plants further confirmed the stable integration of the transgene in T_2_ generation (ESM Fig. S2b). In total, 13 homozygous lines [6 lines from BD-33 (ESM Table 2), four lines from BD-38 and three lines from BD-45] were identified in the T_2_ generation based upon their PCR analysis and the 1:0 segregation pattern of the *AtDREB1A* gene. The harvested T_3_ seeds from T_2_ homozygous lines (from all three events) were advanced further and the gene inheritance and homozygosity confirmed by PCR (ESM Fig.S2c). The selected homozygous lines were analysed for gene expression and in evaluation studies against drought stress in the T_3_ generation.

### Initial screening for drought tolerance at hydroponic stage

Ten 4-week-old plants from each of the five T_3_ homozygous lines (BD-45-3-1, BD-45-10-1, BD-33-24-1, BD-33-24-2 and BD-38-33-1) along with the nontransformed controls were subjected to 20 % PEG treatment in Yoshida’s culture medium to study the response to drought stress. Of these five lines, BD-33-24-1 and BD-33-24-2 showed good growth without wilting, whereas the control plants showed no growth and wilted badly (ESM Fig. S3). Based on these results, we selected lines from the BD-33-24 series for generation advancement. Thirty T_3_ seeds were sown from each of the 15 lines of the BD-33-24 series (from BD-33-24-1 to BD-33-24-15) and transplanted into pots for the dry–down experiment in the biosafety glasshouse.

### Evaluation of T_3_ homozygous lines against drought stress

#### Drought tolerance at vegetative stage

A set of 50 plants at the tenth leaf stage from five different lines (6, 6, 6, 6 and 6 plants from the BD-33-24-4, BD-33-24-5, BD-33-24-6, BD-33-24-7 and BD-33-24-9 lines, respectively; 20 plants from other than the BD-33-24 series) were subjected to the dry–down treatment consisting of the withholding of water for 14 days; another set of 50 plants were maintained as unstressed. During this experiment, physiological parameters, such as chlorophyll content, free proline content, RWC and ion leakage were analysed in transgenic rice lines and nontransgenic controls (both in stressed and unstressed conditions). Under the condition of withdrawing of water, on the fourth day onwards control plants started wilting, whereas transgenic plants did not show any symptoms of wilting and were similar to the unstressed plants. On the 14th day of the experiment, 33 transgenic plants from among the 50 of all of the above-mentioned lines exhibited a very high level of tolerance: the leaves were very healthy and showed no wilting and drying even at the maximum level of drought stress. The morphology of all stressed plants is presented in detail in Table 1. Seventeen transgenic plants showed a moderate level of tolerance, with the leaves showing very little drying and wilting on the 14th day. However, after re-watering for 5 days, all highly tolerant and moderately tolerant plants recovered up to 90–100 %, whereas the control plants started wilting and drying from treatment day 4 onwards and ultimately dried up after 2 weeks (ESM Fig. S4).

**Table 1:** Morphology of T_3_ transgenic and control plants during the dry–down experiment

Sample no.	Line number	Average tiller number	Leaf rolling on treatment day 7	Leaf drying on treatment day 7	Leaf rolling on treatment day 14	Leaf drying on treatment day 14	Recovery after 7 days (%)	Observed phenotype
1	BD-33-24-4	10	H	N	H	N	90–100	HT
2	BD-33-24-5	12	H	N	H	N	90–100	HT
3	BD-33-24-6	15	H	N	H	N	90–100	HT
4	BD-33-24-7	10	H	N	H	N	90–100	HT
5	BD-33-24-9	10	H	N	H	N	90–100	HT
6	BD-33-29-1	9	D	T	H	L	70–89	MT
7	BD-33-30-1	10	D	T	H	L	70–89	MT
8	BD-33-34-1	10	D	T	H	L	70–89	MT
9	BPT Control	12	T	A	C	A	0	SC

*H* Healthy, *T* tightly rolled, *L* leaf margin touching, *D* deep V shape rolling, *N* no drying, *A* apparently dead, *t* tip drying started, *C* completely wilted, *L* little drying, *HT* highly tolerant, *MT* moderate tolerant, *SC* susceptible

Phenotyping carried out as per the standard evaluation system of rice at IRRI on 6 plants per line

#### Confirmation of gene expression under drought stress

Leaf samples from the transgenic rice T_3_ lines (BD-33-24-4 and BD-33-24-5, each 7 plants) were collected on days 3, 6, 9, 12 and 14 of the water stress treatment at regular intervals and RT-PCR analysis was carried out. *AtDREB1A* gene expression was confirmed by RT-PCR analysis; gene expression was very low on day 3 of the water stress treatment but showed continuous increases thereafter, ultimately reaching the highest level on day 15 of the water stress treatment, as evidenced by the amplicon size of 336 and 524 bp with reference to *actin* and *DREB* primers, respectively (Fig. 2c).

### Analysis of physiological parameters

Based on the results of the PCR analysis and the hydroponic experiment described above, T_3_ segregant lines from the BD-33-24 series were selected for the analysis of all physiological parameters under stressed and unstressed conditions. During the dry–down experiment, the lines for analysis in the physiological assays were further selected based on their morphological features, such as tillering mass, leaf rolling, drying, among others. Since the measurement of all these physiological experiments is a destructive process (more leaves should be used), it was carried out in both transgenic and control plants that had an abundance of tillers and healthy leaves.

**Fig. 3:**
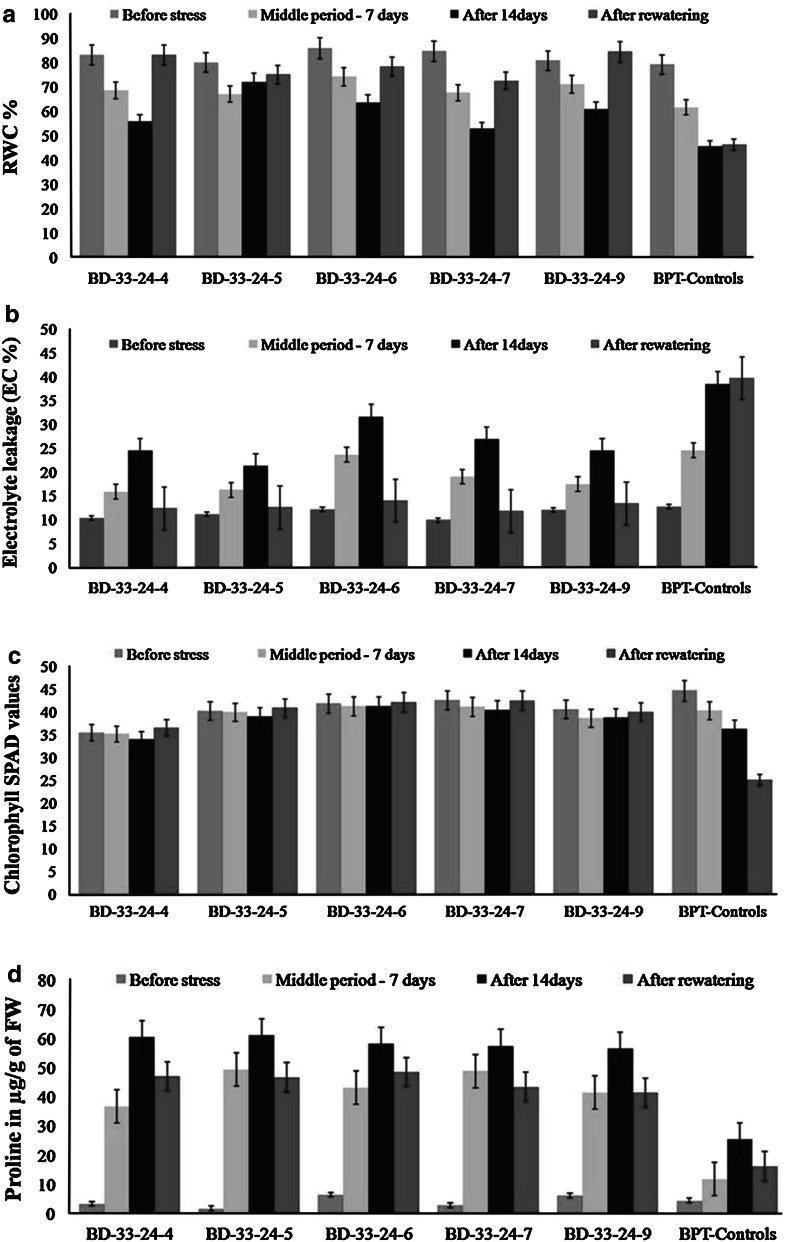
**a** Percentage relative water content (*RWC %*) values of transgenic T_3_ lines and control plants in the water stressed condition [mean values of 6 plants per line: BD-33-24-4 lines (4-3, 4-4, 4-5, 4-6, 4-9, 4–10), BD-33-24-5 lines (5-3, 5-4, 5-7, 5-8, 5-9, 5-10), BD-33-24-6 lines (6-3, 6-4, 6-7, 6-8, 6-9, 6-10), BD-33-24-7 lines (7-3, 7-4, 7-7, 7-8, 7-9, 7-10) and BD33-24-9 lines (9-1, 9-2, 9-7, 9-8, 9-9, 9-10)]. **b** Percentage electrolyte conductivity (*EC %*) values of transgenic T_3_ lines and control plants in the water stressed condition [mean values of 6 plants per line: BD-33-24-4 lines (4-3, 4-4, 4-5, 4-6, 4-9, 4-10), BD-33-24-5 lines (5-3, 5-4, 5-7, 5-8, 5-9, 5-10), BD-33-24-6 lines (6-3, 6-4, 6-7, 6-8, 6-9, 6-10), BD-33-24-7 lines (7-3, 7-4, 7-7, 7-8, 7-9, 7-10) and BD33-24-9 lines (9-1, 9-2, 9-7, 9-8, 9-9, 9-10)]. **c** Chlorophyll values (based on soil and plant analysis device,* SPAD*) of transgenic T_3_ lines and control plants in the water stressed condition [(mean values of 6 plants per line: BD-33-24-4 lines (4-3, 4-4, 4-5, 4-6, 4-9, 4-10), BD-33-24-5 lines (5-3, 5-4, 5-7, 5-8, 5-9, 5-10), BD-33-24-6 lines (6-3, 6-4, 6-7, 6-8, 6-9, 6-10), BD-33-24-7 lines (7-3, 7-4, 7-7, 7-8, 7-9, 7-10) and BD33-24-9 lines (9-1, 9-2, 9-7, 9-8, 9-9, 9-10)]. **d** Proline content of transgenic T_3_ lines and control plants in stressed condition [mean values of 6 plants per line: BD-33-24-4 lines (4-3, 4-4, 4-5, 4-6, 4-9, 4-10), BD-33-24-5 lines (5-3, 5-4, 5-7, 5-8, 5-9, 5-10), BD-33-24-6 lines (6-3, 6-4, 6-7, 6-8, 6-9, 6-10), BD-33-24-7 lines (7-3, 7-4, 7-7, 7-8, 7–9, 7-10) and BD33-24-9 lines (9-1, 9-2, 9-7, 9-8, 9-9, 9-10)].* FW* Fresh weight

RWC was measured before and after subjecting the transgenic rice plants and controls to the water stress treatment. Before stress, there were no obvious differences in the leaf RWC between control and transgenic plants, and the RWC was within the range of 85–95 %. After the plants had been subjected to water stress for 7 days, the RWC of the nontransformed control leaves fell quickly with respect to their first reading (before stress) from 81 to 63 %, whereas the RWC of most of the transgenic plants declined very slowly (BD-33-24-4, 69 %; BD-33-24-5, 68 %; BD-33-24-6, 75 %; BD-33-24-7, 69 %; BD-33-24-9, 72 %. After 14 days of drought stress, the RWC of the transgenic plants had declined by just 15–30 % as compared to 42 % in the control plants. Lines BD-33-24-4, BD-33-24-5, BD-33-24-6, BD-33-24-7 and BD-33-24-9 showed very impressive RWC on day 14, with RWC percentages of 55.0, 75.5, 65.0, 53.0 and 63.0, respectively (Fig. 3a). The rapid decline of the RWC (minimum being 47.0 %) was observed in the control plants after 14 days. Five days after re-watering, the RWC of the most of the transgenic plants recovered up to 79–89 %, which was significantly higher than that observed in the control plants (48 %) whose leaves had almost dried out. However, there was no significant change in the RWCs of the transgenic plants and controls in the unstressed condition.

The drought stress treatment could lead to membrane damage of rice leaves, and ion leakage is the best index of leaf membrane damage which we was measured using an electro-conductivity meter. Initially under unstressed conditions, The EC values of both control and transgenic plants were similar, ranging from 10.5 to 12.5 %. After 7 days of drought stress, ion leakage of the control plants rapidly increased to 25 %; in contrast, in each of the transgenic lines the EC values only slowly increased (BD-33-24-4, 17.5 %; BD-33-24-5, 18.0 %; BD-33-24-6, 24 %; BD-33-24-7, 20 %; BD-33-24-9, 19 %). However, after 14 days of drought stress, electrolyte leakage of the transgenic lines BD-33-24-4, BD-33-24-5, BD-33-24-6, BD-33-24-7 and BD-33-24-9 was only at 25.2, 22.0, 33.0, 30.0 and 27.0 %, respectively, while the control plants showed a very high percentage of ion leakage (41 %; Fig. 3b). It is very clear from Fig. 3b that the difference in the values of the three stages is much lesser in the case of transgenic plants when compared to BPT controls (very high). Moreover, the electrolyte leakage percentage of transgenic plants fell to the primary level after re-watering (BD-33-24-4, 12.5 %; BD-33-24-5, 13.0 %; BD-33-24-6, 15 %; BD-33-24-7, 12.5 %; BD-33-24-9, 15 %) while in the control plants it remained high even after 5 days of re-watering (43 %). The EC values of control plants and transgenic plants in the unstressed condition were almost equal and no ion leakage was observed.

**Fig. 4:**
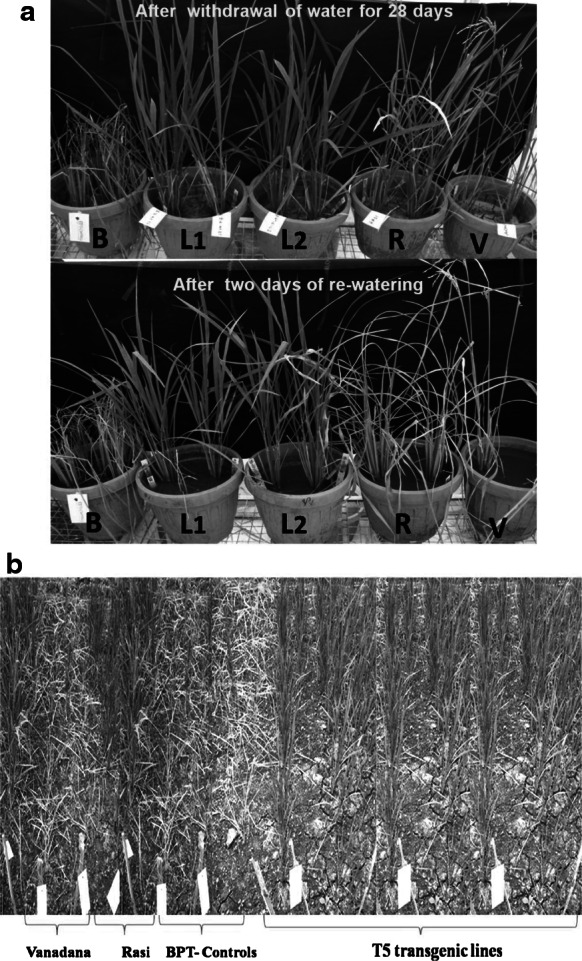
**a** Evaluation of homozygous transgenic lines (T_5_) with nontransgenic drought-tolerant checks Rasi and Vandana in the vegetative stage. *B* BPT controls, *L1* BD-33-24-4-3-1-2, 4, *L2* BD-33-24-4-9-3-4 and-5, *R* Rasi, *V* Vandana. **b** Drought stress screening of T_5_ transgenic lines in real time conditions in the biosafety screenhouse. Transgenic lines (from *left* to *right*) BD-33-24-4-3-1, BD-33-24-4-3-3, BD-33-24-4-3-6, BD-33-24-4-9-3 and BD-33-24-4-9-10

Chlorophyll content is usually measured using a SPAD meter. Transgenic and control plants were measured for their chlorophyll content before and after water stress. Before stress, the SPAD values of the transgenic and nontransgenic control plants were similar, ranging from 35 to 45. After commencement of the drought stress treatment, the chlorophyll values of the control plants showed a very drastic change (30–35) compared to the first reading; in contrast, most of the transgenic plants maintained the same chlorophyll content (40–45) even on day 14 of the stress (Fig. 3c). However, after re-watering for 2 days, transgenic plants showed increased levels of chlorophyll whereas the controls did not.

Proline, which is a common compatible osmo-protectant, accumulates in response to drought stress. During the drought stress treatment, the proline level in both the transgenic and control plants rose continuously. This phenomenon was especially significant after 7 days of stress. After 14 days of stress treatment, transgenic lines BD-33-24-4 (62 μg/g of FW), BD-33-24-5 (63 μg/g of FW) and BD-33-24-6 (60 μg/g of FW) had a twofold higher content of proline, followed by lines BD-33-24-7 (59 μg/g of FW) and BD-33-24-9 (58 μg/g of FW), whereas the control plants showed the lowest level of proline (25 μg/g of FW) (Fig. 3d). There was no difference in the proline contents of unstressed transgenic and control plants.

### Drought tolerance at the flowering stage

After the severe drought stress treatment of transgenic homozygous (T_3_) lines in the vegetative stage, we selected 12 lines (ESM Fig. S5) based on their impressive physiological performance and phenotypic characteristics during the drought stress treatment for testing in the reproductive (anthesis stage) stage by withdrawal of water for 14 days. After commencement of the drought stress, control plants started drying and wilting from day 3 onwards; in contrast, the transgenic lines were healthy (ESM Fig. S6) and no damage to spikelets or pollen grains was observed on day 14. Re-watering for 2 days resulted in the recovery of transgenic plants, but controls became completely dried up after 7 days of drought stress. The spikelet fertility of the stressed transgenic lines BD-33-24-4, BD-33-24-6 and BD-33-24-9 was the highest (60.4, 63.2 and 69.0 %, respectively) when compared to the controls (29.5 %). Similarly, grain yield measurement in these lines also showed (BD-33-24-9) a higher grain yield per plant than controls (15.3 g for BD-33-24-4line, 11.5 g for BD-33-24-6 line, 11.4 g for BD-33-24-9 line and 5.2 g for BPT controls) (ESM Table 3). No significant divergence in grain yield and spikelet fertility was detected between transgenic lines and control plants under unstressed conditions. The spikelet fertility percentages of the unstressed transgenic lines ranged from 64.7 to 74.1 %, whereas that of unstressed controls was 63.0 %. The grain yield of unstressed transgenic lines ranged from 15.9 to 17.1 %, but in controls it was observed as 13.5 % (ESM Table 3). However, the phenology of the stressed transgenic lines was different from that of the unstressed transgenic plants. There was a delay about 15–20 days in seed setting or maturity of the stressed plants compared to the unstressed transgenic and controls.

### Evaluation of drought-tolerant transgenic lines (T_5_) with nontransgenic drought-tolerant check varieties

We have studied the drought tolerance levels of the selected 12 T_5_ lines (10 plants from each line) in the vegetative stage and compared these with the drought tolerance levels of nationally used drought-tolerant check varieties, such as Rasi, Vandana and N-22, by subjecting all plants, in pots, to severe drought stress through the withdrawal of water for a continuous period of 4 weeks (28 days). Most of the transgenic lines were healthy, greenish and showed a greater tolerance for up to 4 weeks compared to the nontransgenic drought-tolerant checks (Fig. 4a). After 28 days, all nontransgenic and WT controls were badly wilted and had dried up. However, after 2 days of re-watering all of the transgenic lines achieved 90–100 % recovery, while the nontransgenic controls (BPT 5204), Rasi, Vandana and N-22 were unable to recover and completely dried. Of 120 T_5_ plants, 60 plants showed 28 days of tolerance, 30 plants showed 21 days of tolerance and the remaining 30 plants showed 15 days of tolerance. These lines also showed a greater tolerance in the booting (ESM Fig. S7a) and anthesis (ESM Fig. S7b) stages for up to 15 days, whereas the controls died 6 days after the withdrawal of water. Another set of 24 T_5_ lines were subjected to severe drought stress treatment for 30 days along with Vandana, Rasi and the WT controls under real time conditions in the biosafety screenhouse. All transgenic lines were greenish, without any wilting or drying, whereas the WT controls, Vandana and Rasi plants had completely dried up after 30 days (Fig. 4b).

## Discussion

Drought is a major constraint affecting rice production, especially in rainfed areas across Asia and sub-Saharan Africa. India accounts for the largest share (59 %) of the total drought-prone rice area in Asia, and most of these areas are rainfed. As drought tolerance is a very complex characteristic, breeding rice varieties with improved drought resistance has progressed slowly over the past few decades, mainly due to a lack of understanding of the traits and genetic mechanisms that confer adaptation to water deficit in the different environments and to the inability of breeders to select for these traits. A few major quantitative trait loci for drought stress have been identified recently and introgressed into the upland varieties. The development of rice varieties with increased tolerance to drought, by both conventional and molecular breeding methods and by genetic engineering, is an important strategy to meet global food demands in a world with less water. Considerable progress has been made in our understanding of gene expression, transcriptional regulation and signal transduction in plant responses related to drought (Zhu et al. 2010). On the other hand, molecular and genomic analyses have facilitated gene discovery (Abe et al. 2003; Seki et al. 2001, 2007; Tran et al. 2004) and enabled genetic engineering approaches to be used to activate or repress several specific functional or regulatory genes or broad pathways related to drought tolerance in plants (Trujillo et al. 2009). Over/inducible expression of the genes that regulate the transcription of a number of downstream drought-responsive genes seems to be a better approach to developing drought-resistant/tolerant transgenic plants when compared to engineering individual functional genes (Bartels and Hussain 2008). Since drought mainly depends on the ecosystem of the upland or lowland rice in terms of its severity and randomness, rice breeders facing major challenges in their attempts to improve drought tolerance in rice varieties due to the diversified rainfed and variable environment conditions across the world. In our study, we attempted to improve drought stress tolerance of the* indica* rice variety Samba Mahsuri (BPT 5204), which is a premium variety having excellent cooking quality and high marketability. We used the TF *AtDREB1A* from *Arabidopsis* to transform this variety, which binds to dehydration responsive element (*DRE/CRT*), a *cis*-acting promoter element which plays an important role in regulating its downstream target gene expression in response to stress in an abscisic acid (ABA)-independent pathway.

The successful transformation and subsequent regeneration of transgenic rice plants using *Agrobacterium*-mediated methods are heavily dependent on many factors, including the type of explants, nutritional supplements, culture conditions prior to and during inoculation, composition of the medium used, duration of co-cultivation, virulence of *Agrobacterium* strain, concentration and composition of the antibacterial agent used, washing of callus, duration of selection and concentration of plant selection agents, conditions of tissue culture, including a robust system of plant regeneration; all are of critical importance (Mohanty et al. 2002; Yookongkaew et al. 2007). In rice, a considerable difference exists between* indica *and *japonica* types in terms of response to callus induction, plant regeneration and transformation (Hiei et al. 1997). Tissue culture and genetic transformation in *japonica* rice has been well studied when compared to* indica* types (Lin and Qifa 2005; Yookongkaew et al. 2007;Yukoh and Toshihiko 2006). The genotypic influence can be overcome by modifying the nutrient medium or transformation conditions, since the same nutrient medium is not ideal for all varieties (Ge et al. 2006). Therefore, we established an efficient transformation and regeneration system for the highly recalcitrant* indica* rice cultivar Samba Mahsuri (BPT-5204) by modifying the 2,4-D concentration in the callus induction medium and NAA concentration in the regeneration medium. The addition of AS in both the pre-culture and co-cultivation medium has been reported to induce *Vir* genes and extend host range of some *Agrobacterium* strains and found to be essential for rice transformation (Saharan et al. 2004). Our observations also confirmed these findings, and the addition of AS in liquid (100 μM) and solid (200 μM) medium was found to be more reliable for transformation with the LBA4404 strain which is a low in virulent strain. Our observations are consistent with a number of previous reports of rice transformation (Kumar et al. 2005). Various researchers have used 50 mg/l hygromycin B for selecting the putative transformants of different* indica*-type rice genotypes (Kant et al. 2007; Sridevi et al. 2005). In our study, we used the same concentration of hygromycin for selecting the callus for three 15-day cycles. We regenerated many putative transgenic plants and obtained three independent events confirmed by PCR and Southern blot analyses. The confirmed transgenic plants did not show any phenotypic changes, such as growth retardation or stunted growth, and were similar to the WT control plants. Oh et al. (2005) suggested that rice is more tolerant in the evolutionary context to the expression of stress-regulated genes than dicots, including *Arabidopsis*. However, plants developed with the *OsCDPK*, *Ubi1:CBF3* and *Ubi1: ABF3* genes also did not exhibit either growth inhibition or visible phenotypic alterations in rice, despite constitutive expression of the transgenes (Oh et al. 2005; Saijo et al. 2000). Similar results were also observed using the stress-inducible *rd29A* promoter in transgenic *Arabidopsis*, tobacco (Kasuga et al. 2004) and rice (Datta et al. 2012). We selected independent single-copy events (Fig. 1d) and advanced to T_3_, T_4_, and T_5_ generations to obtain homozygous lines. The advancement of progenies was based on the results of PCR analysis—not by hygromycin selection—in order to confirm the presence or absence of the transgene by analysing the exact segregation ratio of the integrated transgene *AtDREB1A* (whether 3:1 or approx. 3:1). Although the expression pattern was similar in the three events (BD-33, BD-38 and BD-45) in the T_0_ generation, based on the RT-PCR results, their real osmotic tolerance was different in the homozygous T_3_ lines under the physiological drought induced by the PEG treatment in the hydroponic experiment (ESM Fig. S3). The BD-33 event T_3_ lines performed showed better stress tolerance than the BD-38 and BD-45 lines, thereby confirming the differential expression pattern of the three independent insertional events. The selected BD-33-24 T_3_ homozygous lines were subjected to drought stress in two important stages, namely, the vegetative and reproductive stages. To confirm gene expression under drought stress we performed RT-PCR analyses and observed increasing gene expression with increasing severity of the stress. Gene expression was highest on day 15 of the stress treatment, suggesting that the expression of *AtDREB1A* was under the strict control of the strong inducible *rd29A* promoter.

From the physiological perspective, leaf chlorophyll content has a relationship with photosynthetic rate. The decrease in chlorophyll SPAD values under drought stress could be considered as a typical symptom of oxidative stress and may be the result of pigment photo-oxidation and chlorophyll degradation (Farooq et al. 2009). We recorded very high chlorophyll SPAD values among all of the transgenic plants, and their leaves were more greenish than those of the control plants, thereby confirming normal photosynthesis in transgenic plants.

The process of osmolyte solute accumulation under drought stress is known as osmotic adjustment, and it strongly depends on the rate of plant–water stress. Among these solutes, proline is the most widely studied because of its considerable importance in stress tolerance. Proline accumulation is the first response of plants exposed to a water-deficit condition in order to reduce injury to cells. It affects protein salvation, maintenance of quaternary structure of complex proteins and membrane integrity under dehydration stress and it lowers the oxidation of lipid membranes or photoinhibition (Demiral and Turkan 2004). We observed that transgenic rice lines accumulated highest levels of proline on day 14 of the stress, whereas the WT control plants had very low levels of proline at this time point. The increased proline levels in the transgenic plants indicate that proline accumulation could be one of the factors responsible for tolerance to drought stress. Lines BD-33-24-4 and BD-33-24-5 had the longest healthy period among the transgenic plants, possibly due to their higher level of proline accumulation. However, although proline accumulation under drought stress could be one of the factors responsible for the tolerance, whether the transgene *AtDREB1A* positively regulates the native *P5CS* gene (responsible for proline accumulation) as its downstream gene has yet to be determined.

EC percentage values are the direct indices of ion leakage from stressed plant cells, and it has been reported that the maintenance of their stability and integrity of cell membranes under drought stress is the major component of drought tolerance (Sullivan and Ross 1979). Our study reveals that the cell membrane in most of the transgenic plants was highly intact, i.e., it was stable, compared to that of the controls. RWC is considered to be a measure of plant water status, reflecting the metabolic activity in tissues, and is used as the most meaningful index of water stress tolerance (Nayyar and Gupta 2006). RWC is related to water uptake by the roots as well as water loss by transpiration. During transpiration, stomata closure has been observed as a result of reduced water loss in transgenic plants and is considered to be a characteristic feature of enhanced water stress tolerance (Datta et al. 2012; Pardo 2010). A decrease in the RWC in response to drought stress has been noted in wide variety of plants, as reported by Nayyar and Gupta (2006): when leaves are subjected to drought, leaves exhibit large reductions in RWC and water potential. In our study, RWC percentage values confirmed that the transgenic drought-tolerant lines could retain more water than the controls during the peak stage of the water deficit condition. Moreover, our analysis of gene expression and RWC were done in same leaf; therefore, we can suggest that *AtDREB1A* strongly regulates the drought-responsive genes in our transgenic lines.

Apart from the physiological parameters, gene expression, as evidenced by RT-PCR analysis, and our phenotypic observations of transgenic plants clearly demonstrate that expression of the TF *AtDREB1A* under the control of the stress-inducible promoter *rd29A* substantially elevated drought stress tolerance in transgenic lines in the both vegetative and reproductive stages. A large number of studies have been performed on the characterization and expression of *CBF* genes from different plant species using *Arabidopsis* and other model plants, such as rice and tobacco. Many DREB type genes from different sources have been overexpressed in various crop plants to improve drought tolerance, including *MbDREB1* in *Arabidopsis* (Yang et al. 2011), *AtDREB1A* in *Lolium perenne* (Li et al. 2011), *TsCBF1* in maize (Zhang et al. 2010), *OsDREB1F* in* Arabidopsis* (Wang et al. 2008) and *OsDREB1B* in tobacco (Gutha and Reddy 2008). In rice, various genes of the DREB1 class (from both rice and *Arabidopsis*), such as *OsDREB1A*, *OsDREB1B*, *OsDREB1Ci*, *AtDREB1A*, *AtDREBIB* and *AtDREB1C*, have been overexpressed under the control of either drought-responsive promoters, such as *rd29A* and *OsHVA22p*, or various constitutive promoters (Chen et al. 2008; Hsieh et al. 2002; Ito et al. 2006; Oh et al. 2005; Xiao et al. 2009; Yang et al. 2010). The gene *AtDREB1A* has also been successfully introduced in various crops for improving drought tolerance, such as wheat (Pellegrineschi et al. 2004), tobacco (Kasuga et al. 2004), tall fescue (Zhao et al. 2007), bahiagrass (James et al. 2008), groundnut (Vadez et al. 2007), peanut (Bhatnagar-Mathur et al. 2007), maize (Al-Abed et al. 2007) and soya bean (Paiva Rolla et al. 2013). However, the development of *DREB*-transformed transgenic plants in different rice genotypes with strong tolerance to drought is still in the experimental phase. Drought phenotyping to identify high-performance lines under realistic greenhouse screening is essential due to the restrictions on open field trials involving transgenic material for biosafety issues. Several of the studies conducted on transgenic crops to date have achieved successful transgene expression, but may have given rise to misleading conclusions from an agronomic or physiological perspective (Bhatnagar-Mathur et al. 2008) mainly due to the fact that testing transgenic lines is generally conducted under artificial stress conditions, in small pots and at early seedling growth stages (Yang et al. 2010). In our study, the evaluation of transgenic lines was based on the high survival of transgenic plants after severe water deficit stress under greenhouse conditions. It is clear from the results shown in Fig. 4a that transgenic lines L1 (BD-33-24-4-3-1-2, 4) and L2 (BD-33-24-4-9-3-4, 5) were more healthy after 28 days of severe stress than after 2 days of re-watering, indicating that the drought-inducible expression of the *AtDREB1A* gene driven by the strong promoter *rd29A* functions in a drought-tolerant pathway. It has been suggested that this improved survival of the transgenic plants after severe drought conditions could be associated with either the activation of genes related to drought resistance or a more conservative growth pattern in the transgenics compared with controls (Bhatnagar-Mathur et al. 2008; Morran et al. 2011). The activation of genes in transgenic plants may involve the stress-inducible genes, such as *LEA/COR/DHN* genes, and is yet to be determined.

The improvement of drought tolerance in rice genotypes should be achieved without a parallel limitation of plant growth and yield potential (Cattivelli et al. 2008). In our study, we demonstrated that stress-inducible expression of the *AtDREB1A* TF in rice improved the drought tolerance in association with grain yield under bipartite conditions, i.e. stressed and unstressed conditions (ESM Table 3). Based on our observations, a number of factors contributed to the improved grain yield of transgenic rice compared to the WT controls under drought conditions. Firstly, the transgenic lines were more tolerant to drought because they had elevated levels of proline compared to the WT controls which provided protection from osmotic damage (Fig. 3d). Secondly, transgenic lines had a higher number of tillers, lower degree of leaf rolling and drying, quicker recovery percentages (Table 1) and larger panicle sizes than controls, all factors which directly contribute to grain yield. Most importantly, the flag leaf is thought to make the greatest contribution to spikelet fertility, thereby increasing grain filling (ESM Table 3), compared with the other leaves of the same plant (Chen et al. 2007; Mahmood et al. 1991). It is reasonable to predict that grain yield can be substantially improved if the photosynthetic capacity of the flag leaves is raised. Transgenic rice had a larger flag leaf area (ESM Fig. S4) than control plants which significantly increases the photosynthetic rate and net photosynthate level, thus increasing the grain yield. Furthermore, pollen viability of transgenic plants was high when compared to control plants (data not shown). Similarly, transgenic plants under unstressed conditions also showed a significant higher grain yield than control plants due to the contribution of better agronomical characters, such as an increased number of tillers and panicles, larger panicle sizes, increased spikelet fertility (ESM Table 3) and higher pollen viability (data not shown). Since transgenic plants have undergone tissue culture cycle, it is presumed that there could be a positive pleiotropic effect, resulting in the improved agronomic and morphological traits in transgenic plants under unstressed conditions.

Datta et al. (2012) reported that a knowledge of the effects of drought treatment in the pre-flowering and post-flowering stages was essential to determine the dehydration effects on flowering and seed development. In our study, the T_5_ individual sub-lines of BD-33-24-4-10 were used to separately check for drought tolerance (but not the same plants; ESM Fig. S7) at the booting (L1: BD-33-24-4-10-1-7,8, 9 and L2: BD-33-24-4-10-3-1,2, 3) and anthesis (L3: BD-33-24-4-10-4-1,2, 3 and L4: BD-33-24-4-10-5-2,3, 4) stages. Hence, all of these lines were tolerant at either the booting or anthesis stage up to 14 days, and no sterility was observed in any line, apart from their normal spikelet fertility and grain yields (similar to their parents in the earlier generations T_3_ and T_4_) which were higher in the transgenic plants than in the WT control plants. Spikelet fertility, grain filling and grain yield of the T_5_ transgenic plants were also clearly significantly higher than those of the WT control plants in unstressed conditions (data not shown).

Although there has been considerable progress in developing transgenic rice, their effective phenotyping for improving drought tolerance under field conditions remains to be assessed. Most of the researchers evaluated transgenic plants by subjecting them to the stress at the seedling stage for periods ranging from 1 h to few days in small containers; this represents a ‘shock’ treatment and provides only unrealistic conditions (Babu et al. 2004; Rabbani et al. 2003). Hence, it is important to conduct experiments under real time conditions that will help researchers to closely estimate the drought tolerance in the field in terms of timing, duration and severity (Hu et al. 2006). However, from an agricultural point of view, drought occurs simultaneously with other abiotic stresses, and the evaluation of transgenic lines in the field or under field-like conditions is essential (Deikman et al. 2011; Peleg et al. 2011; Varshney et al. 2011). In another experiment, we imposed severe water stress alone (no other abiotic stresses) on transgenic plants continuously for 4 weeks (28 days) in pots and as well as in the screenhouse under biosafety real time conditions (Fig. 4b) which approximately resembled the stress condition in the field. Transgenic plants showed greater drought tolerance and a more rapid recovery upon re-watering when compared with nontransgenic and WT controls such as Rasi, Vandana, N-22 and BPT5204. Drought phenotyping of transgenic lines in pots and under real time conditions resulted in the selection of the best events in terms of tolerance among the same individual lines. However, as reported earlier by Datta et al. (2012), variation in level of tolerance among the same individual lines was observed, possibly due to the differential regulation of downstream genes by the *AtDREB1A* TF in each individual plant, which is to be confirmed by the microarray analysis.

The improvement of drought stress tolerance is a major goal of genetic engineering in rice, and our results have confirmed that stress-inducible expression of the *AtDREB1A* gene in transgenic plants greatly enhanced drought tolerance at both the vegetative and reproductive stages without jeopardizing the physiological responses of the plants. The *Arabidopsis* gene *CBF3/DREB1A* functioned in the stress tolerance pathway and substantially enhanced drought stress tolerance in highly susceptible* indica* rice cultivar Samba Mahsuri without affecting its agronomic traits. Since this variety is a premium variety with high marketability—but susceptible to drought stress—the selected transgenic lines would be a valuable resource and can be used as a pre-breeding and direct variety for the improvement of rice under water stress conditions.

## Electronic supplementary material

Below is the link to the electronic supplementary material.

Supplementary material 1 (DOCX 8720 kb)

Supplementary material 2 (DOCX 15 kb)
