# Supplementary material for: Stress-inducible expression of AtDREB1A transcription factor greatly improves drought stress tolerance in transgenic indica rice
Source: Transgenic Res. 2014 Jan 8;23(3):421–39. doi: 10.1007/s11248-013-9776-6 (PMC4010723; doi:10.1007/s11248-013-9776-6)
Supplement: Supplementary file 1 — Supplementary material 1 (DOCX 8720 kb) [file 11248_2013_9776_MOESM1_ESM.docx]

**Figure: S1**

**
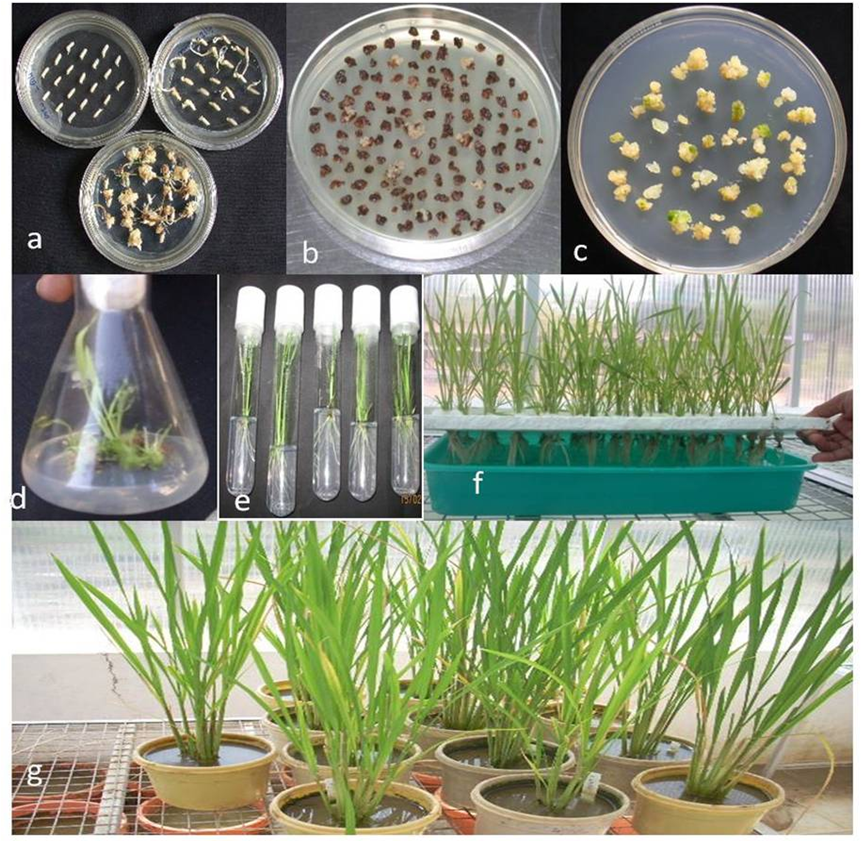
**

**Figure S1:** a) Seed inoculation and callus induction; b) Selection of transformed calli in hygromycin medium; c) Antibiotic resistant calli in regeneration medium; d) Regeneration of green plantlets; e) Fully grown plants with well developed roots; f) Hardening of plants in Yoshida’s culture medium; g) Primary transgenic plants (T_0_) grown in pots in bio-safety glass house.

**Figure: S2**

a


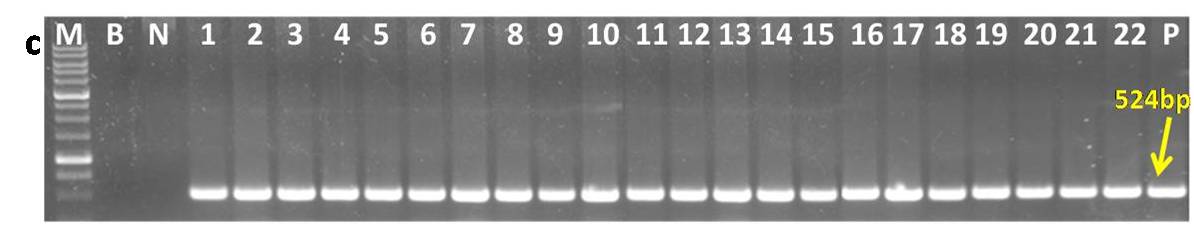


**Figure S2a:** PCR analysis of T_2_ BPT-DREB1A plants of BD-33-24 sub lines with gene specific primers: M: 1Kb DNA ladder, B: Blank, N: Non transformed control plant, Lanes 1-22: T_2_ DREB plants (BD-33-24-1 to BD-33-24-22) and P: Positive control (plasmid).


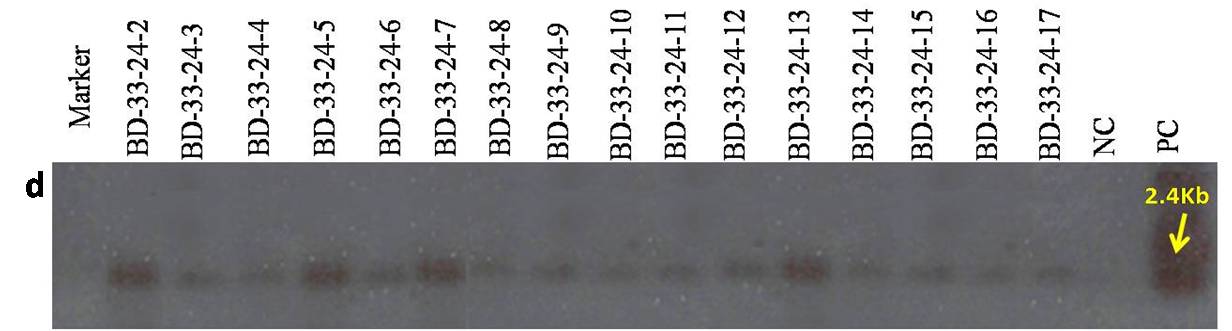


b

**Figure S2b**) Southern analysis of T_2_ BPT-DREB1A plants of BD-33-24 sub lines with plasmid probe: Marker: Lamda/HindIII digest, BD33-24-2 to BD33-24-17 T_2_ plants of BD-33-24 line, NC: Non transformed control plant and PC: Positive control (2.4kb eluted fragment of gene expression cassette)


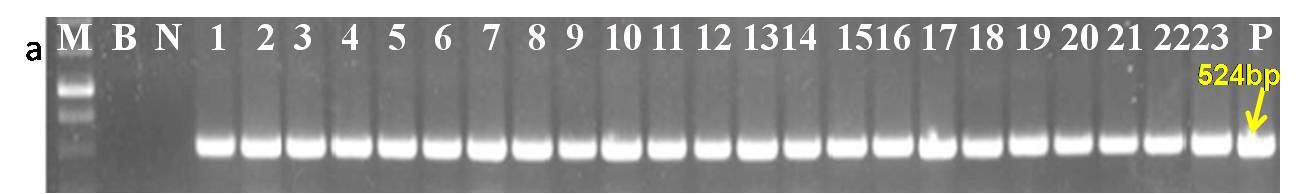


c

**Figure S2c**) PCR analysis of T_3_ lines of BD-33-24 lines: M: 1Kb ladder, B: Blank, N: Non transgenic control plant, Lanes 1-23 T_3_ DREB plants; Lane1 to Lane 6: BD-33-24-4 lines (4-3, 4-4, 4-5, 4-6, 4-9 & 4-10), Lane7 to Lane 12: BD-33-24-5 lines (5-3, 5-4, 5-7, 5-8, 5-9 & 5-10), Lane13 to Lane18: 33-24-6 lines (6-3, 6-4, 6-7, 6-8, 6-9 & 6-10), Lane19 to Lane23: BD-33-24-7 lines (7-3, 7-4, 7-7, 7-8, 7-9 & 7-10) and P: Positive control (plasmid).

**Figure: S3**

**
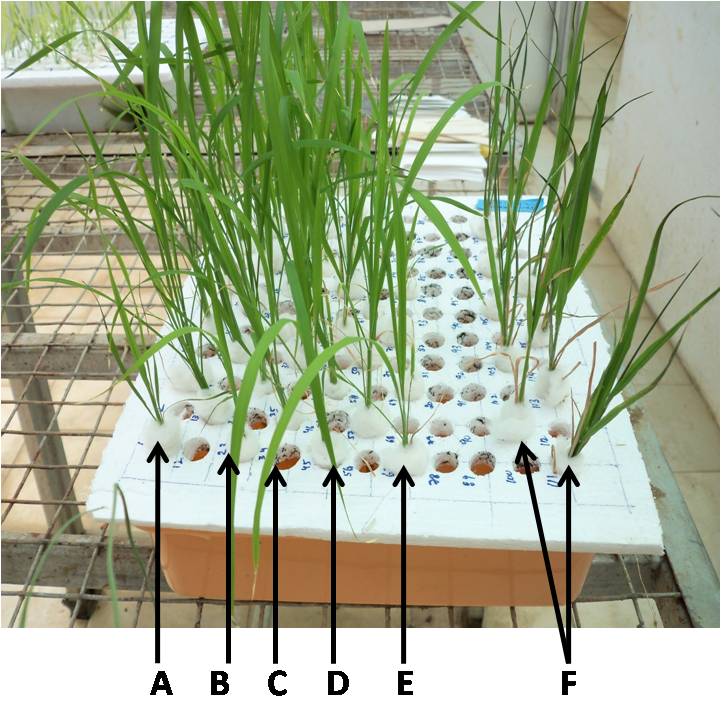
**

**Figure S3:** Hydroponic study of four week old homozygous transgenic rice lines (T_3_ generation) with 20% PEG for drought stress: A) BD-45-3-1, B) BD-45-10-1, C) BD-33-24-1, D) BD-33-24-2, E) BD-38-33-1 and F) BPT controls.

**Figure: S4**

**
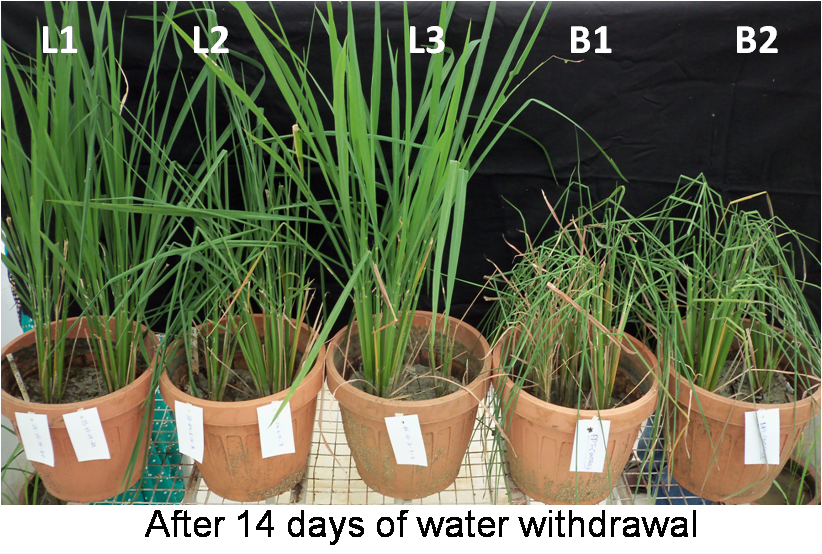
**

**Figure S4:** Survival of T_3_ transgenic plants after 14 days of stress treatment in vegetative stage: L1, L2, & L3: BPT Transgenic rice lines; B1 & B2: Non transgenic control plants (BPT 5204)

**Figure S5**

**
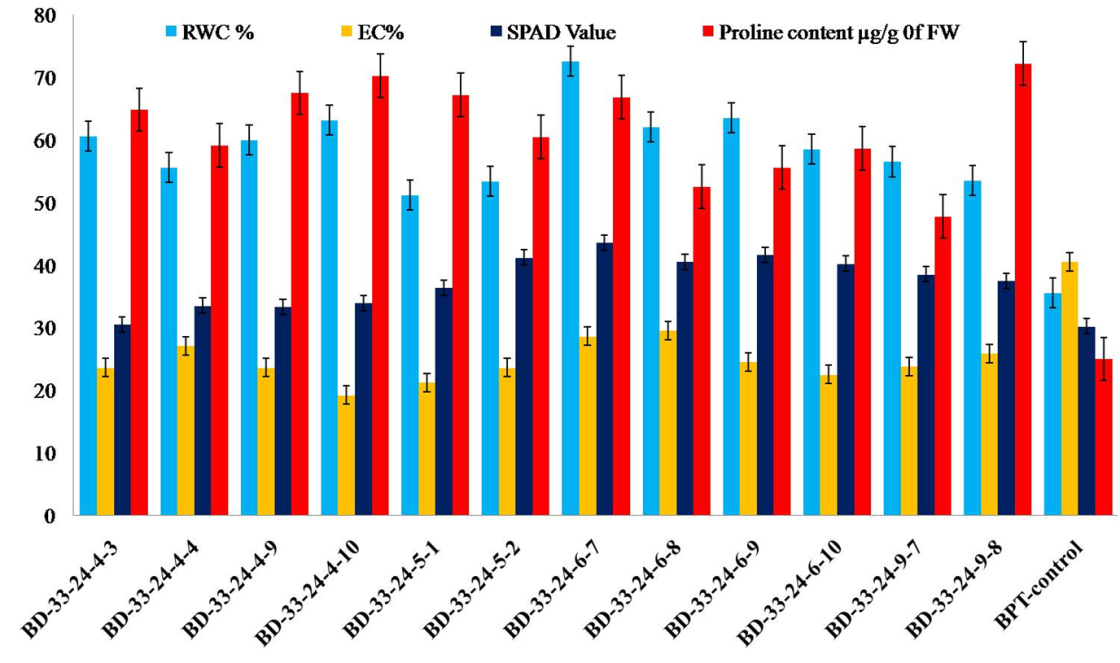
**

**Figure S5:** Study of various physiological parameters of BPT transgenic rice lines at T_3_ generation.

**Figure S6**


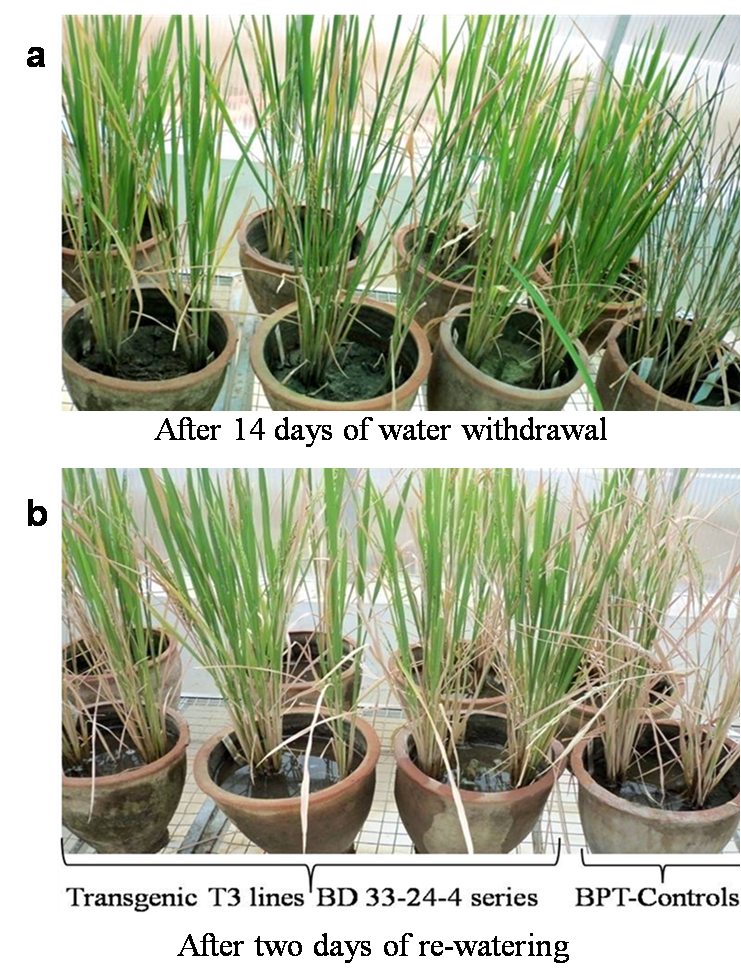


**Figure S6:** Drought stress treatment of T_3_ lines in reproductive stage: a) after withdrawal of water for 14 days b) after two days of re-watering.

**Figure S7**


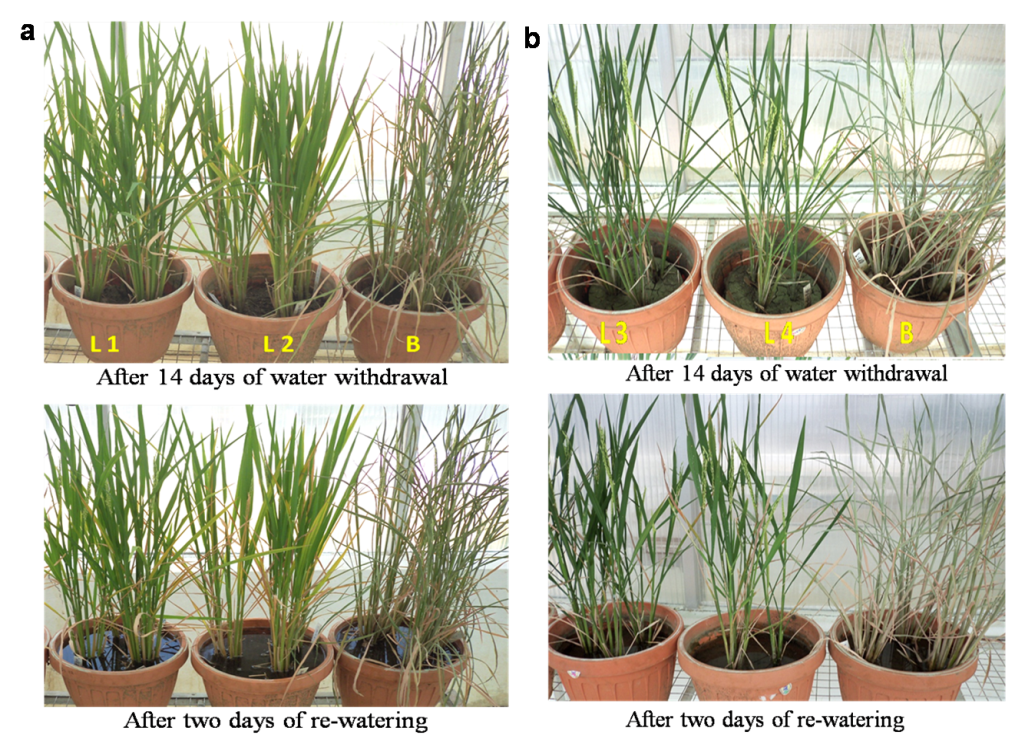


**Figure S7: a)** Evaluation of T_5_ transgenic lines in booting stage (pre flowering stage): L1: BD-33-24-4-10-1-7, 8 & 9, L2: BD-33-24-4-10-3-1, 2 &3 and B: BPT controls **b**) Evaluation of T_5_ transgenic lines in anthesis stage: L3: BD-33-24-4-10-4-1,2&3, L4: BD-33-24-4-10-5-2, 3 &4 and B: BPT controls.
