# Supplementary material for: Stress-inducible expression of AtDREB1A transcription factor greatly improves drought stress tolerance in transgenic indica rice
Source: Transgenic Res. 2014 Jan 8;23(3):421–39. doi: 10.1007/s11248-013-9776-6 (PMC4010723; doi:10.1007/s11248-013-9776-6)
Supplement: Supplementary file 2 — Supplementary material 2 (DOCX 15 kb) [file 11248_2013_9776_MOESM2_ESM.docx]

**Supplementary Tables**

Table -1: Molecular and segregation analysis of T_1_ transgenic plants*

| Line No. | No of seeds raised | No of plants obtained | No of PCR +ves | No of PCR -ves | Ratio | No of Southern +ves | Chi square test | P value |
| --- | --- | --- | --- | --- | --- | --- | --- | --- |
| BD-33 | 50 | 42 | 32 | 10 | 3:1 | 32 | 0.032 | 0.8586 |
| BD-38 | 50 | 40 | 26 | 14 | ~3:1 | 26 | 2.133 | 0.1441 |
| BD-45 | 50 | 40 | 30 | 10 | 3:1 | 30 | 0.000 | 1.000 |

* PCR was taken as basis for segregation analysis and only both PCR, Southern positive plants were advanced for next generation

Table-2: Segregation pattern of BD-33 lines in T_2_ generation*

| S. No | Plant Identity | Chi square value | P value | Ratio of PCR  positive: Negative | Homozygous/  Heterozygous |
| --- | --- | --- | --- | --- | --- |
| 1 | BD33-8 | 4.813 | 0.0282 | 19:6 | He |
| 2 | BD33-10 | 4.813 | 0.0282 | 18:7 | He |
| 3 | BD33-15 | 8.333 | 0.0039 | 25:0 | H |
| 4 | BD33-16 | 8.333 | 0.0039 | 25:0 | H |
| 5 | BD33-17 | 8.333 | 0.0039 | 25:0 | H |
| 6 | BD33-18 | 8.333 | 0.0039 | 25:0 | H |
| 7 | BD33-20 | 0.013 | 0.9081 | 19:6 | He |
| 8 | BD33-23 | 4.813 | 0.0282 | 14:11 | He |
| 9 | BD33-24 | 8.333 | 0.0039 | 25:0 | H |
| 10 | BD33-29 | 8.333 | 0.0039 | 25:0 | H |
| 11 | BD33-31 | 7.053 | 0.0079 | 13.12 | He |
| 12 | BD33-32 | 1.613 | 0.2040 | 16.9 | He |

* 25 plants were raised from each line, H- Homozygous, He- Heterozygous

Table-3: Comparison of spikelet fertility and grain yield between T_3_ transgenic and control plants under stressed and unstressed conditions.

| Line No. | Spikelet fertility % | | Grain yield (gms) | |
| --- | --- | --- | --- | --- |
|  | unstressed | stressed | unstressed | stressed |
| BD-33-24-4 | 69.4 | 60.4 | 17.1 | 15.3 |
| BD-33-24-5 | 68.0 | 53.2 | 16.1 | 11.2 |
| BD-33-24-6 | 64.7 | 63.2 | 15.9 | 11.5 |
| BD-33-24-7 | 67.5 | 65.2 | 16.3 | 11.6 |
| BD-33-24-9 | 74.1 | 69.0 | 16.8 | 11.4 |
| BPT controls | 63.0 | 29.5 | 13.5 | 5.2 |
